# Mechanisms of mesothelial cell response to viral infections: HDAC1-3 inhibition blocks poly(I:C)-induced type I interferon response and modulates the mesenchymal/inflammatory phenotype

**DOI:** 10.3389/fcimb.2024.1308362

**Published:** 2024-02-27

**Authors:** Flavia Trionfetti, Claudia Montaldo, Ivan Caiello, Giulio Bontempi, Michela Terri, Marta Tiberi, Vanessa Marchant, Alessandro Domenici, Paolo Menè, Marco Cordani, Clemens Zwergel, Giusi Prencipe, Marta Ruiz-Ortega, Sergio Valente, Antonello Mai, Marco Tripodi, Raffaele Strippoli

**Affiliations:** ^1^ Department of Molecular Medicine, Sapienza University of Rome, Rome, Italy; ^2^ Gene Expression Laboratory, National Institute for Infectious Diseases, Lazzaro Spallanzani IRCCS, Rome, Italy; ^3^ Division of Rheumatology, Ospedale Pediatrico Bambino Gesù IRCCS, Rome, Italy; ^4^ Cellular Biology in Renal Diseases Laboratory, IIS-Fundación Jiménez Díaz-Universidad Autónoma Madrid, Madrid, Spain; ^5^ 15 REDINREN/RICORS2040, Madrid, Spain; ^6^ Renal Unit, Department of Clinical and Molecular Medicine, Sant’Andrea University Hospital, Sapienza University of Rome, Rome, Italy; ^7^ Department of Biochemistry and Molecular Biology, Faculty of Biology, Complutense University of Madrid, Madrid, Spain; ^8^ Instituto de Investigaciones Sanitarias San Carlos (IdISSC), Madrid, Spain; ^9^ Department of Drug Chemistry and Technologies, Sapienza University of Rome, Rome, Italy

**Keywords:** mesothelial cells, HDAC, viral infections, MMT, interferon response, inflammatory cytokines

## Abstract

Infectious peritonitis is a leading cause of peritoneal functional impairment and a primary factor for therapy discontinuation in peritoneal dialysis (PD) patients. Although bacterial infections are a common cause of peritonitis episodes, emerging evidence suggests a role for viral pathogens. Toll-like receptors (TLRs) specifically recognize conserved pathogen-associated molecular patterns (PAMPs) from bacteria, viruses, and fungi, thereby orchestrating the ensuing inflammatory/immune responses. Among TLRs, TLR3 recognizes viral dsRNA and triggers antiviral response cascades upon activation. Epigenetic regulation, mediated by histone deacetylase (HDAC), has been demonstrated to control several cellular functions in response to various extracellular stimuli. Employing epigenetic target modulators, such as epidrugs, is a current therapeutic option in several cancers and holds promise in treating viral diseases. This study aims to elucidate the impact of TLR3 stimulation on the plasticity of human mesothelial cells (MCs) in PD patients and to investigate the effects of HDAC1-3 inhibition. Treatment of MCs from PD patients with the TLR3 agonist polyinosinic:polycytidylic acid (Poly(I:C)), led to the acquisition of a bona fide mesothelial-to-mesenchymal transition (MMT) characterized by the upregulation of mesenchymal genes and loss of epithelial-like features. Moreover, Poly(I:C) modulated the expression of several inflammatory cytokines and chemokines. A quantitative proteomic analysis of MCs treated with MS-275, an HDAC1-3 inhibitor, unveiled altered expression of several proteins, including inflammatory cytokines/chemokines and interferon-stimulated genes (ISGs). Treatment with MS-275 facilitated MMT reversal and inhibited the interferon signature, which was associated with reduced STAT1 phosphorylation. However, the modulation of inflammatory cytokine/chemokine production was not univocal, as IL-6 and CXCL8 were augmented while TNF-α and CXCL10 were decreased. Collectively, our findings underline the significance of viral infections in acquiring a mesenchymal-like phenotype by MCs and the potential consequences of virus-associated peritonitis episodes for PD patients. The observed promotion of MMT reversal and interferon response inhibition by an HDAC1-3 inhibitor, albeit without a general impact on inflammatory cytokine production, has translational implications deserving further analysis.

## Introduction

Infectious peritonitis is one of the most serious complications of PD, leading to fibrosis and functional damage (Mehrotra et al., 2016). Medical practices such as catheter positioning and dwelling, as well as abdominal surgery, can facilitate peritoneum infection. Peritonitis is often driven by skin gram-positive bacteria and, less frequently, by enteric flora gram-negative bacteria. Instead, there are limited reports on the role of viruses. In 20% of the cases, the cultures from peritonitis result in negative results, and in these cases, it is possible to hypothesize a viral pathogenesis (Aufricht et al., 2017).

MCs are leading players in the development of serosal inflammation and fibrosis during infections because they synthesize components of the extracellular matrix protein (ECM) such as fibronectin-1 (FN-1), collagens, as well as mediators of inflammation such as prostaglandins and prostacyclin, cytokines, and chemokines (Strippoli et al., 2016; Trionfetti et al., 2023b). Invading microorganisms induce the first phase of peritoneal inflammation characterized by the resident macrophages and MCs, whose cross-talk plays a role in the amplification of the inflammatory response through the production of proinflammatory cytokines (Jonjic et al., 1992; Topley et al., 1993a; Topley et al., 1993b; Terri et al., 2021). Clinical studies on PD dialysis fluid from PD patients with peritonitis identified a specific “fingerprint” to diagnose bacterial infection based on the cytokine produced (Lin et al., 2013).

MCs sense microbial derivatives by innate pattern recognition receptors (PRRs), and among them, TLRs have a main role (Takeuchi and Akira, 2010). The decoy effect of soluble TLRs recognizing bacterial components such as sTLR2 has been analyzed in their ability to reduce inflammation and peritoneal fibrosis (Raby et al., 2017; Raby et al., 2018).

Thus, while several studies have been performed based on models of peritonitis induced by bacterial/fungal infections, the impact of viral infections on MC physiopathology has been far less characterized (Hurst et al., 2001).

Chemical covalent modifications of histone proteins drive the shift from heterochromatin to euchromatin and vice versa, regulating gene expression in eukaryotic cells (Mai, 2010; Ferguson et al., 2011; Zwergel et al., 2015). In particular, increased lysine acetylation at the histone level is generally associated with the transcriptional activation of genes, whereas hypoacetylation is linked to hampered transcription, thus silencing genome regions. Acetylation and deacetylation levels of histones, as well as nonhistone proteins, are tightly controlled and fine-tuned by two key opponent enzyme families, histone acetyltransferases (HATs) and histone deacetylases (HDACs). Both families are part of larger multiprotein complexes comprising various other proteins correlated with transcriptional activation/repression. Up to now, 18 distinct human HDACs have been discovered and subsequently divided into four classes (I–IV) considering their sequence homology to the HDACs of *Saccharomyces cerevisiae* (RPD3, HDA1, and SIR2) (Lakshmaiah et al., 2014; Di Bello et al., 2022). Class I HDAC expression is increased in both tumor and nontumor inflammatory/profibrotic conditions, including peritoneal fibrosis (Hayashi et al., 2010; Nural-Guvener et al., 2014; Zhou et al., 2023).

Moreover, several reports demonstrated that HDACs are implicated in the pathogenesis of viral infections (Herbein and Wendling, 2010; Panella et al., 2016; Trionfetti et al., 2023a). Lipopolysaccharide (LPS) stimulation was demonstrated to enhance HDAC3 activity, leading to tumor necrosis factor-α (TNF-α) production (Zhu et al., 2010). Treatment with modulators of epigenetic targets, i.e., epidrugs, is a current therapeutic option in several cancers and could represent an approach in the therapy of viral diseases. HDAC inhibitors (HDACis) are a group of natural or newly synthesized compounds able to block HDAC activity, restoring or increasing histone acetylation levels.

Indeed, pharmacological inhibition of HDAC activity is a current therapeutic option in cancer management and could represent a promising approach in viral diseases (Panella et al., 2016). To date, several trials are ongoing using HDACis, as identified by ClinicalTrials.gov and discussed by Bondarev et al. (2021).

Entinostat (MS-275) is a synthetic benzamide specific for class I HDAC, and it is a selective inhibitor for HDAC1-3 (Saito et al., 1999; Khan et al., 2008). MS-275 activity has been analyzed, especially in a frame of therapy of leukemias and solid tumors. However, its use to limit organ fibrosis has been demonstrated to be protective in urinary bacterial infections (Lyu et al., 2019; Schwartz et al., 2023).

The aim of this study is to characterize the response of MCs from PD patients to viral infections mimicked by TLR3 stimulation in terms of inflammatory/profibrotic response and subsequently analyze the role of HDAC1-3 inhibition.

We found that treatment of MCs with Poly(I:C) induced a bona fide MMT characterized by the downregulation of epithelial genes and the acquisition of a mesenchymal-like phenotype. Moreover, inflammatory cytokine production was increased. Treatment with MS-275 promoted the reacquisition of epithelial-like features and the inhibition of an interferon (IFN) response, but modulated the expression of inflammatory cytokines/chemokines in a nonunivocal manner. Since these drugs are in trial combinations for tumors and are studied in the treatment of fibrosis, these observations deserve further investigation.

## Materials and methods

### Antibodies and chemicals

The primary antibodies used for Western blotting experiments are mAb anti-E-cadherin (BD610181) from BD Transduction Laboratories (Franklin Lakes, NJ, USA). mAbs anti-Mx1 (sc-34128), anti-tubulin (sc-32293), anti-HSP90 (sc-13119) were from Santa Cruz Biotechnology (Dallas, TX, USA); Abs anti-IFIT1 (23247-1-AP), anti-Snail (L70G2), anti-STAT1 (9172), anti-phospho STAT3 (Y701), and anti-STAT3 (9132) were from Cell Signaling Technology (Danvers, MA, USA). pAb anti-IFITM1 (600-74-1) was from Proteintech (Rosemont, IL, USA). pAbs anti-MMP14 (ab53712) was from Abcam (Cambridge, UK): pAbs anti-phospho STAT1 (s727).

HRP-conjugated secondary antibodies used were purchased from Jackson ImmunoResearch, Cambridge, UK: anti-rabbit (JI 711-036-152) and anti-mouse (JI 715-036-150).

Antibodies for immunofluorescence include the following: anti-FN 1 (ABCAM, ab2413) and anti-calnexin (Santa Cruz Biotechnology, sc-23954), Cy3-conjugated anti-rat secondary antibodies (Jackson ImmunoResearch, 112-165-003), and anti-mouse Alexa Fluor 488-conjugated (A32723 Thermo Fischer Scientific, Waltham, MA, USA). DRAQ5 staining solution (No. 130-117-343) was from Miltenyi Biotec, Bergisch Gladbach, Germany. Antibodies for ELISA, including anti-TNF-α, anti-IL-1β, anti-IL-6, anti-CXCL8, and anti-CXCL10, were from R&D Systems (Minneapolis, MN, USA). Poly(I:C) (HMW) was from Invivogen (San Diego, CA, USA). Human TGF-β1 (Cat.100-21C) was from Peprotech (Cranbury, NJ, USA). MS-275 was from Mai Lab.

### Patients and cell culture

Effluent-derived MCs were isolated from 14 PD patients as previously described (Lopez-Cabrera et al., 2006). The demographic and clinical features of the patients are described in **Table 1**. Effluent-derived MCs were cultured in Earle’s M199 supplemented with 10% FBS (Gibco-Life Technologies), 2 mM of l-glutamine (EuroClone, Milan, Italy), 100 U/ml of penicillin, 100 μg/ml of streptomycin (Gibco-Life Technologies), and 2.5 μg/ml of amphotericin B.

**Table 1:** Demographic and Clinical features of the PD patients.

Patient	sex	age	cause of kidney failure	Diabetes	Hypertension	months on PD	PD tecnique	excharges	glucose (mg(dl)	peritonitis	hemoperitoneum	escapes
1	M	56	malignant hypertension	no	yes	40	CAPD	1	2270	no	no	no
2	M	69	unknown	no	yes	44	CAPD	4	1580	no	no	no
3	F	85	p-ANCA vasculitis	no	yes	58	APD	3	1815	no	no	no
4	M	75	Ig AGNF	no	yes	92	CAPD	15 litres/night	1360	no	no	no
5	M	61	diabetes/hypert ension	yes	yes	12	CAPD	1	2270	no	no	yes
6	F	66	p-ANCA vasculitis	no	yes	31	CAPD/CCPD	4/20 litres/24h	2270	no	no	no
7	M	66	Chronic pielonephritis	no	yes	72	CAPD	2	2270	2	no	no
8	M	53	ADPKD	no	yes	6	CAPD	3	2270	2	no	yes
9	M	54	unknown	no	yes	13	CAPD	1	1360	no	no	no
10	M	64	Ig A GNF	no	yes	55	CCPD	25 litres/24h	1580	2	no	yes
11	M	57	type 1 diabetes mellitus	yes	yes	56	CCPD	20 litres/24h	1815	no	no	no
12	M	77	unknown	no	yes	5	CAPD	1	1360	no	no	no
13	F	65	ADPKD	no	yes	11	CAPD	1	2270	no	no	no
14	F	29	extracapillary GNF	no	yes	3	CAPD	1	2270	no		

MCs were grown at 37°C in a humidified atmosphere with 5% CO_2_. MCs from patients were treated when specified with MS-275 (0.25 μM), Poly(I:C) (2 ng/ml), and TGF-β1 (2 ng/ml). Treatment with MS-275 was repeated every 48 h during the experimental procedure.

### MTT assay

To evaluate cell viability upon Poly(I:C) stimulation, primary MCs were stimulated with different doses of Poly(I:C) for 24 h and then tested with the CyQUANT™ MTT Cell Viability Assay (V13154) from Invitrogen (Waltham, MA, USA) following manufacturer instructions. Absorbance was measured at 480 nm with a Microplate Reader from Bio-Rad (Hercules, CA, USA).

### Western blotting

Monolayers of effluent-derived MCs or MeT-5A cells were lysed in CelLytic™ MT Cell Lysis Reagent (Sigma-Aldrich), and proteins were quantified by Bradford protein assay reagent (Bio-Rad).

Gels were electrophoresed at 100 V in a running buffer (25 mM of Tris, 190 mM of glycine; 0.1% SDS) and then transferred to a nitrocellulose or PVDF membranes in a transfer buffer (50 mM of Tris, 40 mM of glycine; 0.1% SDS; 20% methanol). Blots were blocked in 5% nonfat milk prepared in TBS Tween (10 mM of Tris-HCl at pH 7.5; 150 mM of NaCl; 0.05% Tween 20). Nitrocellulose-bound antibodies were detected by chemiluminescence with ECL (Immobilon Western HRP substrate, Millipore, Burlington, MA, Stati Uniti). Blots were acquired with the Chemidoc Touch imaging system and analyzed with Image Lab Software release 6.0 (Bio-Rad Laboratories). Molecular size marker ladder (No. PM2610, Thermo Fisher Scientific).

### Cytokine detection

MCs were left untreated or treated with 0.25 μM MS-275 for 48 h. MCs were then treated with 2 ng/ml Poly(I:C) for 24 h alone or in the presence of MS-275. Supernatants were collected 24 h after treatment. TNF-α, IL-1β, IL-6, CXCL8, and CXCL10 were measured in supernatant samples using an ELISA assay (R&D Systems Inc., MN, USA).

### Gelatine zymography

Equal amounts of proteins were loaded onto a 7.5% SDS polyacrylamide gel containing gelatin (4 mg/ml) (Thermo Fisher Scientific) and electrophoresed for 2 h. The gels were washed two times with renaturing buffer (2.5% Triton X100, 50 mM of Tris HCL at pH 7.5, 5 mM of CaCl_2_, 1 µM of ZnCl_2_). Subsequently, the gels were incubated in an incubation buffer (1% Triton X100, 50 mM of Tris HCL at pH 7.5, 5 mM of CaCl_2_, 1 µM of ZnCl_2_) while gently shaking at 37°C for 16~18 h. The gels were then stained with staining buffer (0.5 g Coomassie blue, 40% methanol, 10% acetic acid, 50% H_2_O) for 30 min while gently shaking. Finally, the gels were destained with destaining buffer (40% methanol, 10% acetic acid, 50% H_2_O). The area of enzymatic activity is characterized by no Coomassie blue staining (white bands).

### Reverse-transcriptase polymerase chain reaction

Cellular RNA was extracted from cell cultures using TRIzol reagent (Life Technologies, Carlsbad, CA, USA) or the RNeasy Mini Kit (Qiagen, Hilden, Germany), according to the manufacturer’s instructions. cDNA synthesis was generated using a reverse transcription kit (A3500) from Promega (Madison, WI, USA), according to the manufacturer’s recommendations. cDNAs were amplified by qPCR reaction using Maxima SYBR Green/ROX qPCR Master Mix (K0253) from Thermo Fisher Scientific (Waltham, MA, USA). qPCR reactions were performed with the Rotor-Gene 6000 thermocycler (Corbett Research, Cambridge, UK). The primer sequences used in this study are shown in **Table 2**. Relative amounts, obtained with the 2 (−ΔCt) method, were normalized with respect to the housekeeping gene L34. Statistical significance was determined with a *t*-test with Prism version 8.0. Differences were considered significant at *p* < 0.05. Values are reported in the graphs.

**Table 2:** List of primers for RT-PCR analysis.

Target gene	Forward Sequence	Reverse Sequence
**hCALB2 **	ATCCTGCCAACCGAAGAGAA	GCCAAGCCTCCATAAACTCG
**hCXCL-8**	AGCCTTCCTGATTTCTGCAGCTCT	AATTTCTGTGTTGGCGCAGTGTG
**hECAD**	TACGCCTGGGACTCCACCTA	CCAGAAACGGAGGCCTGAT
**hFN-1**	GGCTGACAGAGAGAGFTTCCCG	AGCTGGGTCTGCTAACATCAC
**hIFIT1**	CTTCAGGATGAAGGACAGGAAG	ACTTGGCTGCATATCGAAAGA
**hIFITM1**	GAAGTCTAGGGACAGGAAGA	CAGAGCCGAATACCAGTAAC
**hIL-1β**	AGCCATGGCAGAAGTACCTG	CCTGGAAGGAGCACTTCATCT
**hIL-6**	AGTCCTGATCCAGTTCCTGC	CATTTGTGGTTGGGTCAGGG
**hL34**	GTCCCGAACCCCTGGTAATAGA	GGCCCTGCTGACATGTTTCTT
**hMMP14**	TCTGGCGGGTGAGGAATA	CTCTCGTAGGCAGTGTTGATG
**hMMP9**	GCGCTGGGCTTAGATCATT	GCCATTCACGTCGTCCTTAT
**hMx1**	AAGCCTGATCTGGTGGACAAAGGA	AACCCTTCTTCAGGTGGAACACGA
**hSNAIL**	CACTATGCCGCGCTCTTTC	GCTGGAAGGTAAACTCTGGATTAGA
**hTLR1**	CAATGCTGCTGTTCAGCTCTTC	GCCCAATATGCCTTTGTTATCC
**hTLR2**	AATCCTCCAATCAGGCTTCTCT	TGTAGGTCACTGTTGCTAATGTAGGT
**hTLR3**	GAAAGGCTAGCAGTCATCCAAC	GTCAGCAACTTCATGGCTAACA
**hTLR5**	ACAAGATTCATACTCCTGATGCTACTG	CCAGGAAAGCTGGGCAACTA
**hTNFα**	GCTGCACTTTGGAGTGATCG	TCACTCGGGGTTCGAGAAGA

### Immunofluorescence and confocal microscopy

After specific treatments, MCs were fixed for 20 min in 4% formaldehyde in PBS, were permeabilized in 0.2% Triton X-100/PBS for 5 min, and were blocked with 2% BSA for 20 min. Coverslips were mounted in Prolong Gold antifade (Life Technologies) and examined under a confocal microscope (Leica TCS SP2, Wetzlar, Germany). Digital images were acquired with the Leica software, and the image adjustments and merging were performed using the appropriate tools of ImageJ software. A minimum of four fields per sample (at least 150 total cells per total) from two independent experiments was analyzed. ImageJ was used to quantify relative fluorescence.

### Proteomics: protein digestion, peptide purification, and nanoLC analysis

Effluent-derived MCs (*n* = 2) were left untreated or pretreated with 0.25 μM of MS-275 for 48 h. MCs were then treated with 2 ng/ml Poly(I:C) for 24 h alone or in the presence of MS-275. MCs were lysed in RIPA buffer and quantified by Bradford assay. A quantitative mass spectrometry analysis was conducted by Montaldo et al. (2021).

### Statistical analysis

Statistical significance was determined with a *t*‐test using GraphPad Prism version 8.0 (La Jolla, CA, USA). Differences were considered significant at ^*^ *p* < 0.05, ^**^ *p* < 0.01, and ^***^ *p* < 0.001.

Perseus software (version 1.6.7.0), after log2 transformation of the intensity data, was applied to the proteomic study. Statistical analysis was carried out on proteins identified in 70% of the samples. Results were considered statistically significant at *p* < 0.05. To improve visualization, a *z*-score plot and a cluster heat map were generated, and gene ontology enrichment analysis of biological processes, molecular functions, and cellular components was performed.

## Results

### Stimulation with Poly(I:C) increases the expression of TLR3 and other TLRs relevant in the response to pathogens in primary MCs from PD patients

**Figure 1:**
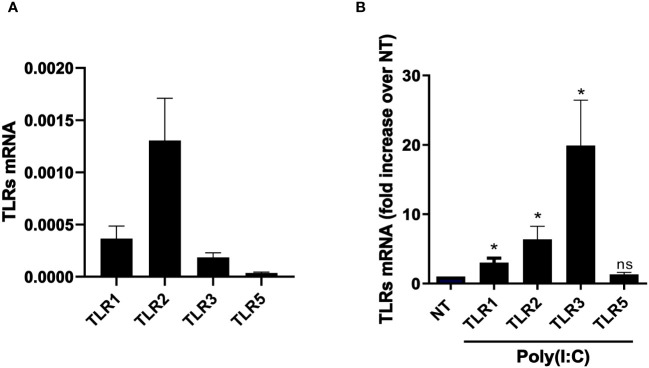
MCs express a specific subset of TLRs, which results in modulation upon Poly(I:C) stimulation. **(A)** Quantitative RT-PCR expression analysis of *TLR1*, *TLR2*, *TLR3*, and *TLR5* in untreated MC primary cells from PD patients (MCs). *L34* mRNA levels were used for normalization. Bars represent the mean ± SEM of triplicate determinations in at least seven independent analyses. **(B)** MeT5A was treated with 2 ng/ml of Poly(I:C) for 24 (h) Quantitative RT-PCR expression analysis of *TLR1*, *TLR2*, *TLR3*, and *TLR5* in Poly(I:C)-treated MCs compared to nontreated samples (NT). *L34* mRNA levels were used for normalization. Bars represent the mean ± SEM of triplicate determinations in at least six independent experiments. *p*-value was calculated with respect to NT samples. Differences were considered significant: ^*^ *p* < 0.05; ns: not significant.

To characterize the response of MCs from PD patients to viral infections, the expression of TLR ligands in primary MCs from PD patients was first analyzed. Untreated MCs were found to express TRL1, TRL2, TRL3, and TLR5 (**Figure 1A**). To set up an experimental system mimicking RNA virus infection, MCs were treated with the TLR3 agonist Poly(I:C), a double-strand synthetic RNA molecule. Cell stimulation with 2 ng/ml of Poly(I:C) was considered appropriate since only a limited decrease in cell vitality (around 10%) was observed at this dose in an MMT assay (**Image 1**).

Interestingly, Poly(I:C) treatment led to an increase of TLR3 and also of TLR1 and TLR2 expression (**Figure 1B**).

### Poly(I:C) stimulation induces MMT

**Figure 2:**
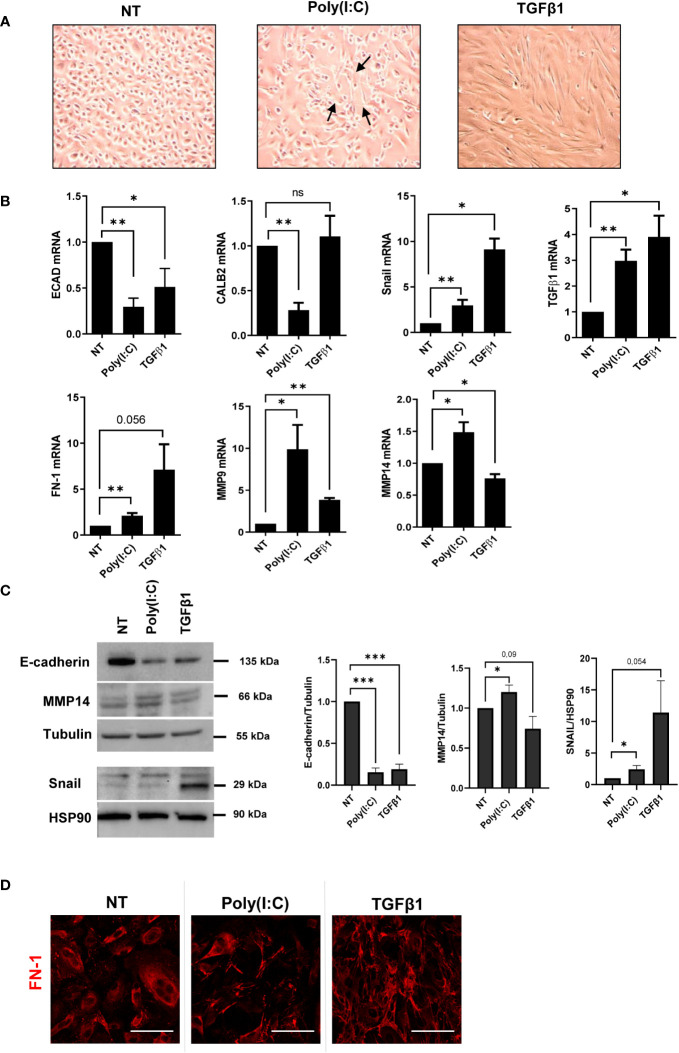
Poly(I:C) induces MMT in MCs. **(A)** MCs were treated with 2 ng/ml of Poly(I:C) or 2 ng/ml of TGF-β1 for 24 (h) MCs after Poly(I:C) or TGF-β1 stimulation compared to NT. Poly(I:C)-stimulated MCs showed a typical spindle-like shape (arrow), indicating the acquisition of a mesenchymal-like morphology. **(B)** MCs were treated with 2 ng/ml Poly(I:C) or 2 ng/ml TGF-β1 for 24 (h) Quantitative RT-PCR expression analysis of *E-CAD*, *CALB2*, *SNAIL*, *TGF-β1*, *FN*, *MMP9*, and *MMP14* in Poly(I:C)- or TGF-β1-treated MCs compared to NT. *L34* mRNA levels were used for normalization. Bars represent the mean ± SEM of triplicate determinations in at least six independent experiments. **(C)** MCs were treated with 2 ng/ml of Poly(I:C) or 2 ng/ml of TGF-β1 for 48 (h) Left: Western blot showing the expression of E-cadherin, MMP14, and SNAIL from total MC lysates. Tubulin and HSP90 were detected as loading controls. Right: WB quantification in Poly(I:C) or TGF-β1-treated MCs compared to NT. Bars represent the mean ± SEM of triplicate determinations in at least four independent experiments. *p*-value was calculated with respect to NT samples. Differences were considered significant: ^*^ *p* < 0.05; ^**^ *p* < 0.01; ^***^ *p* < 0.001; ns, not significant. **(D)** MCs were treated with 2 ng/ml of Poly(I:C) or 2 ng/ml of TGF-β1 for 48 (h) Immunofluorescence of MCs treated with Poly(I:C) or TGF-β1 compared with NT MCs. Fixed cells were stained with antibodies against FN-1. A minimum of 150 cells per sample from three independent experiments were analyzed. Scale bar: 50 μm.

Changes in cellular plasticity leading to the acquisition of a mesenchymal phenotype by MCs were then analyzed. Treatment with Poly(I:C) promoted the induction of a *bona fide* MMT characterized by the acquisition of a spindle-like shape in MCs (**Figure 2A**). Gene expression analysis revealed the downregulation of the epithelial markers E-cadherin and Calretinin and the upregulation of the mesenchymal marker Snail, the EMT master gene, and TGF-β1 (**Figure 2B**).

Moreover, fibrosis-related genes such as FN-1, metalloproteinase (MMP)-9, and MMP-14 were also induced (**Figure 2B**). The expression of E-cadherin, MMP14, and Snail was confirmed by Western blot analysis (**Figure 2C**). Immunofluorescence experiments revealed FN-1 fiber formation upon Poly(I:C) treatment (**Figure 2D**). These results indicated that stimulation with Poly(I:C) is sufficient to induce a *bona fide* MMT in MCs from PD patients.

### Treatment with MS-275 rescues Poly(I:C)-induced MMT

**Figure 3:**
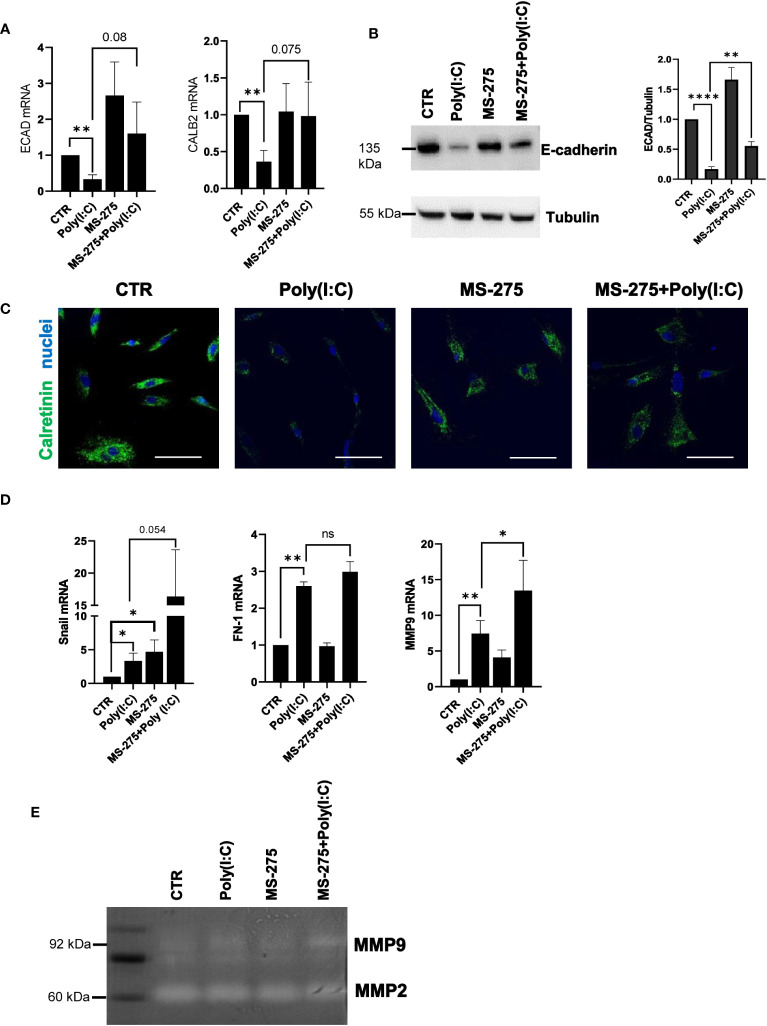
Treatment with MS-275 promotes the reacquisition of epithelial features in Poly(I:C)-treated MCs. MCs were left untreated or treated with 0.25 μM of MS-275 for 48 (h) MCs were then treated with 2 ng/ml Poly(I:C) for 24 h alone or in the presence of MS-275. **(A)** Quantitative RT-PCR expression analysis of *ECAD* and *CALB2* from Poly(I:C) with/or MS-275-treated MCs compared to CTR. *L34* mRNA levels were used for normalization. Bars represent the mean ± SEM of triplicate determinations in at least five independent experiments. *p*-value was calculated with respect to CTR or Poly(I:C) samples. Differences were considered significant: ^*^ *p* < 0.05; ^**^ *p* < 0.01; ^****^ *p* < 0.0001. **(B)** Left: Western blot showing the expression of E-cadherin in the total cellular extract of MCs. Tubulin was detected as a loading control. Right: WB quantification of E-cadherin expression in Poly(I:C) with/or MS-275-treated MCs compared to CTR. Bars represent the mean ± SEM of triplicate determinations in at least four independent experiments. **(C)** Immunofluorescence of MCs treated with Poly(I:C) with/or MS-275 compared with CTR. Fixed MCs were stained with an antibody against calretinin. Nuclei were stained with DRAQ5. A minimum of 150 cells per sample from two independent experiments was analyzed. Scale bar: 50 μm. **(D)** Quantitative RT-PCR of *Snail*, *FN*, and *MMP9* expression from Poly(I:C) with/or MS-275-treated MCs. *L34* mRNA levels were used for normalization. Bars represent the mean ± SEM of triplicate determinations in at least five independent experiments. *p*-value was calculated with respect to CTR or Poly(I:C) samples. Differences were considered significant: *p < 0.05; **p < 0.01; ns, not significant **(E)** Gelatine zymography showing the activity of MMP9 and MMP2 in native protein extracted from MCs treated with Poly(I:C) alone or with MS-275 compared to CTR. The image shows a representative experiment of four performed on four different MC primary samples.

Since previous reports demonstrated the role of HDAC1-3 inhibition by MS-275 in promoting MMT reversal in TGF-β1-treated MCs from PD patients, the effect of MS-275 in Poly(I:C)-mediated induction of a mesenchymal-like phenotype was analyzed (Rossi et al., 2018). In this experimental condition, MS-275 rescued the expression of the epithelial markers E-cadherin and calretinin (**Figures 3A, B**). Protein expression of E-cadherin and calretinin was confirmed by Western blot and confocal microscopy analyses, respectively (**Figures 3B, C**). Interestingly, the mRNA expression of the mesenchymal markers Snail, FN-1, and MMP9 was upregulated upon MS-275/Poly(I:C) treatment (**Figure 3D**). However, Snail was previously demonstrated to lose its repressive activity toward E-cadherin expression in the presence of MS-275 (Rossi et al., 2018). Although MS-275 treatment significantly upregulated MMP9 mRNA expression, this did not correspond to increased activity at a gelatin zymography analysis (**Figure 3E**). Overall, these results demonstrated that MS-275 treatment rescues Poly(I:C)-induced MMT.

### Proteomic analysis of MS-275/Poly(I:C)-treated primary MCs reveals a complex reprogramming of MC proteome

In order to clarify the molecular mechanisms underlying the effect of HDAC1-3 inhibition in this experimental system, the proteome from MCs left untreated, treated with Poly(I:C) alone, or in combination with MS-275 was analyzed by quantitative mass spectrometry analysis.

Following proteolytic digestion, peptides were separated into 8 fractions based on their hydrophobicity before being analyzed by label-free liquid chromatography-mass spectrometry.

**Figure 4:**
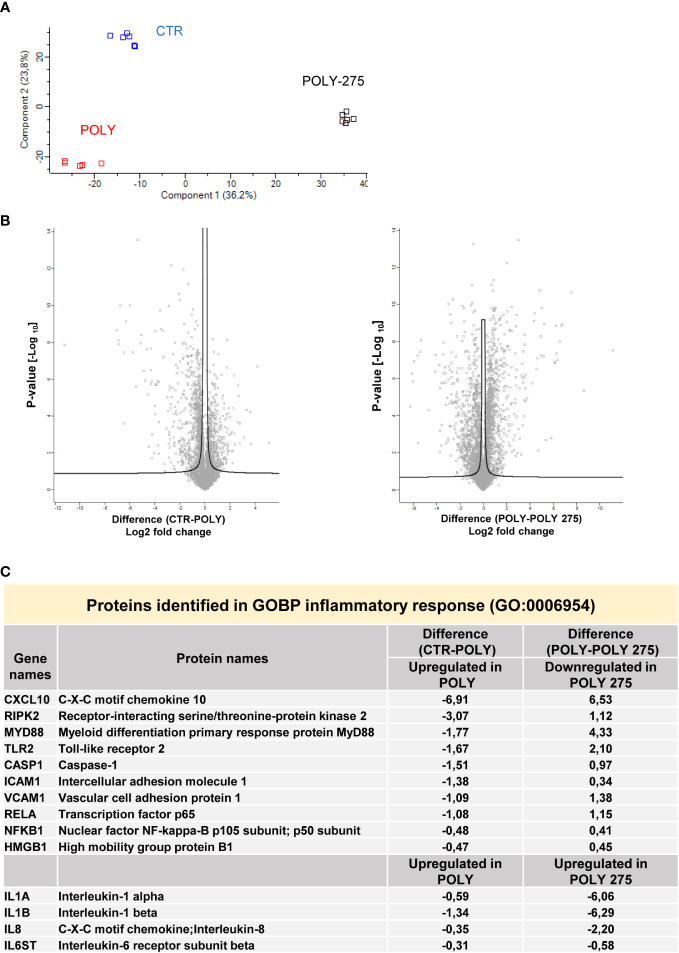
Treatment with Poly(I:C) and MS-275 impacts on the proteome of mesenchymal-like MCs. MCs were left untreated, stimulated with 2 ng/ml Poly(I:C) alone for 24 h or after pretreatment with MS-275 (0.25 μM) for 48 (h) **(A)** PCA of the LFQ intensities obtained in CTR-, Poly-, and Poly-275-treated sample datasets. **(B)** Volcano plots comparing CTR vs. Poly (left panel) and Poly vs. Poly-275 (right panel). Black curves represent the significance threshold at a false-discovery rate (FDR) of 0.05 and S0 of 0.1. **(C)** Table showing selected identified proteins belonging to the GOBP inflammatory response. PCA, principal component analysis; Poly, Poly(I:C); GOBP, gene ontology biological process.

Principal component analysis (PCA) indicated that control MCs are distributed in a distinct group from MCs treated with Poly(I:C) or with MS-275/Poly(I:C) (**Figure 4A**). In differential expression analysis (DEA) comparing samples represented by the Volcano plot (left, control vs. Poly(I:C)-treated MCs; right, Poly(I:C)-treated vs. MS-275/Poly(I:C)-treated MCs), proteins identified were differentially expressed with FDR < 0.05. In total, 737 proteins were found upregulated in Poly(I:C)-treated vs. control MCs; 939 proteins were found downregulated in Poly(I:C)/MS-275 vs. Poly(I:C)-treated MCs (**Figure 4B**, **Data Sheet 1**). Stimulation with Poly(I:C) promoted the induction of inflammatory markers. In particular, molecules relevant for TLR signaling, such as TLR2, RIPK2, MYD88, RELA, and NFKB1 (GOBP: 0006954 inflammatory response), were induced by treatment with Poly(I:C) being inhibited by Poly(I:C)/MS-275 cotreatment. Indeed, some inflammatory cytokines/chemokines, including IL-1α, IL-1β, CXCL8, and IL-6ST, were upregulated by treatment with MS-275 (**Figure 4C**, **Data Sheet 2**).

**Figure 5:**
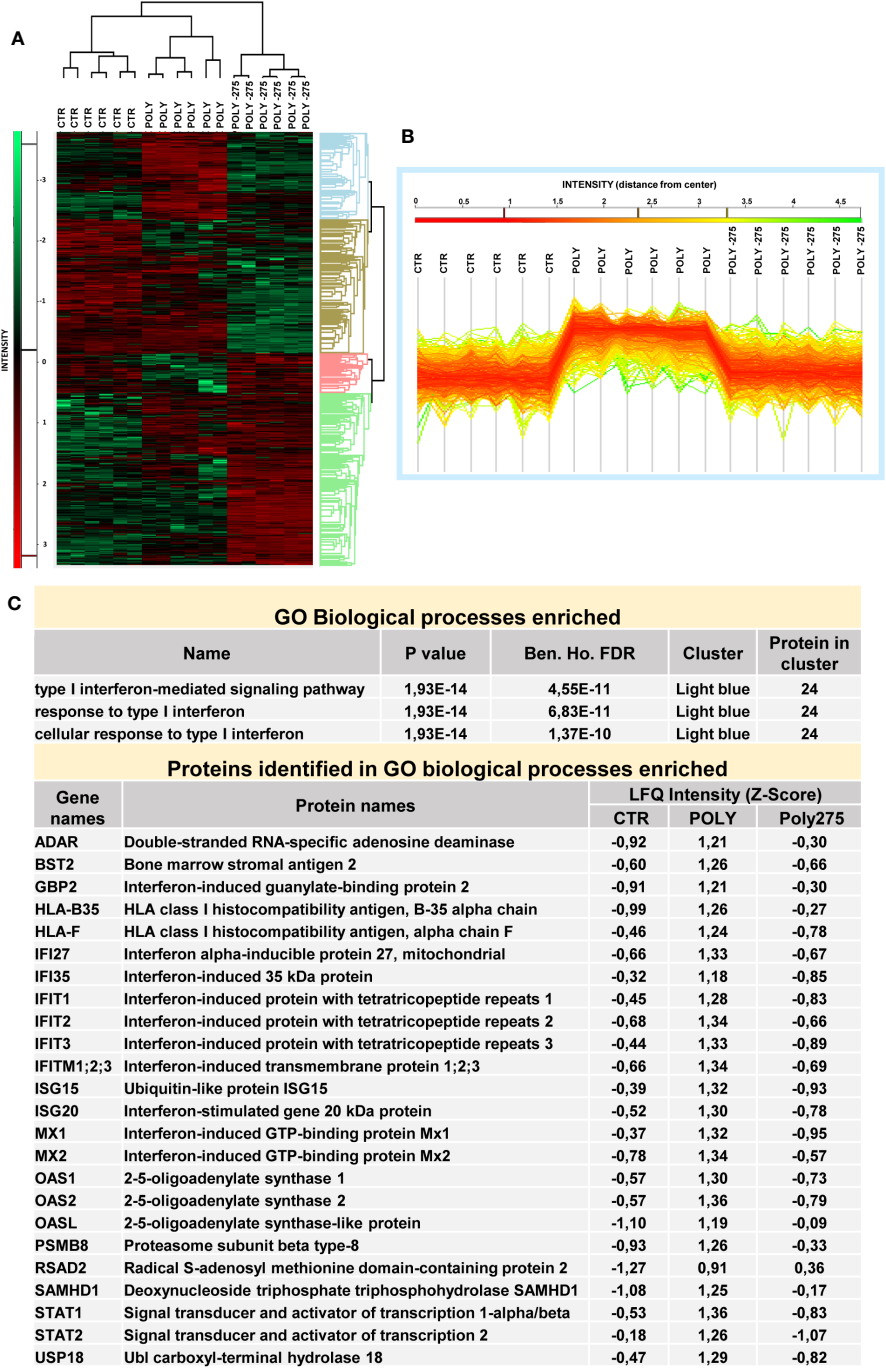
Treatment with MS-275 inhibits a Poly(I:C)-mediated type I-IFN response in mesenchymal-like MCs. MCs were left untreated, stimulated with 2 ng/ml Poly(I:C) alone for 24 h or after pretreatment with MS-275 (0.25 μM) for 48 (h) **(A)** Heat map of differentially expressed proteins in CTR, Poly, and Poly-275 samples. LFQ intensities are expressed in *z*-score values (range of intensity *z*-score: ± 3.5). Up- and downregulated proteins are expressed in red and green scales, respectively. Hierarchical clustering was performed using Euclidean distance and average linkage using the Perseus software. **(B)** Intensity profile plot of the light-blue cluster. **(C)** GO enrichment analysis of the light-blue cluster performed by Perseus software on differentially expressed proteins between the CTR, Poly, and Poly-275 datasets. The table displays GOBP-enriched proteins related to interferon I response and the identified proteins with specific LFQ intensity. Poly, Poly(I:C); GO, gene ontology; GOBP, gene ontology biological process; LFQ, label-free quantification. Abbreviations: Poly (Poly(I:C)) 275 (MS-275).

Hierarchical clustering classified the samples into three groups based on differentially expressed proteins, as represented by heat map visualization (**Figure 5A**). Gene ontology enrichment analysis on the light blue cluster (**Figure 5B**) reveals significant inhibition of Poly(I:C)-induced type I-IFN response upon MS-275 treatment (**Figure 5C**, **Data Sheet 3**).

Overall, while treatment with MS-275 promoted a general inhibition of the IFN response induced by Poly(I:C), the modulation of the inflammatory markers and inflammatory cytokine production was not univocal.

### Inhibition of Poly(I:C)-induced type-I interferon response by MS-275 is associated with reduced STAT1 tyrosine phosphorylation

**Figure 6:**
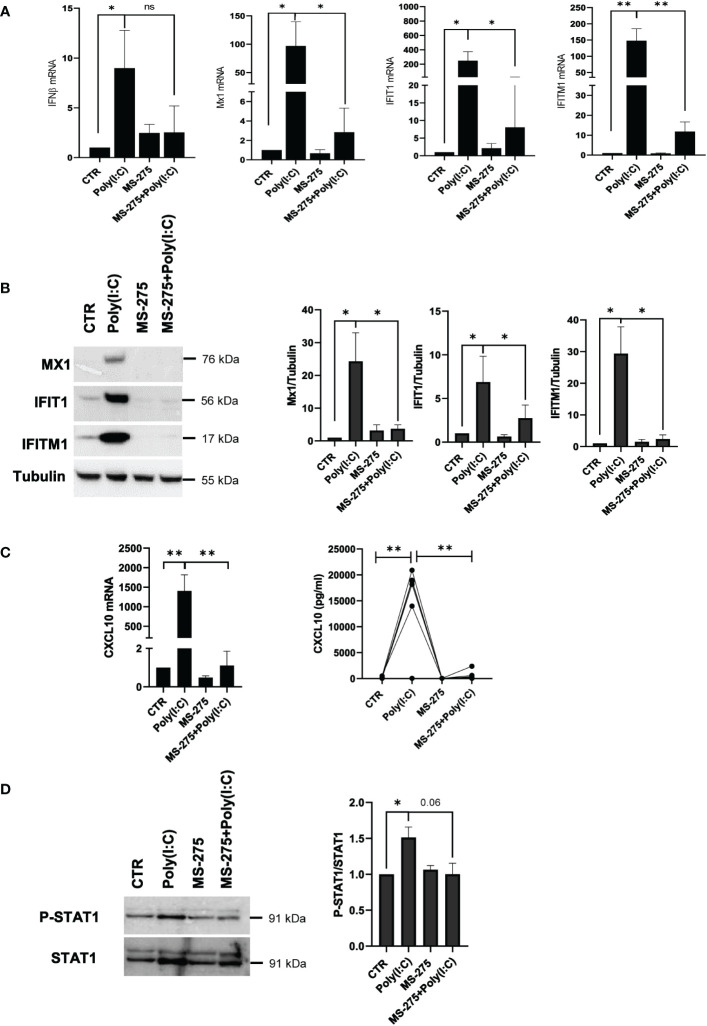
MS-275 inhibits a Poly(I:C)-mediated type I-IFN response and STAT1 tyrosine phosphorylation in mesenchymal-like MCs. MCs were left untreated or pretreated with 0.25 μM of MS-275 for 48 (h) MCs were then treated with 2 ng/ml Poly(I:C) for 24 h alone or in the presence of MS-275. **(A)** Quantitative RT-PCR of *IFN-β*, *Mx1*, *IFIT1*, and *IFTM1* expression in Poly(I:C) with/or MS-275-treated MCs. *L34* mRNA levels were used for normalization. Bars represent the mean ± SEM of triplicate determinations in at least seven independent experiments. *p*-value was calculated with respect to CTR or Poly(I:C) samples. **(B)** Left: Western blot showing the expression of Mx1, IFIT1, and IFITM1 in the total cellular extract of MCs. Tubulin was detected as a loading control. Right: WB quantification of Mx1, IFIT1, and IFITM1 expression in Poly(I:C) with/or MS-275-treated MCs compared to CTR. Bars represent the mean ± SEM of triplicate determinations in at least four independent experiments. *p*-value was calculated with respect to CTR or Poly(I:C) samples. **(C)** Left: Quantitative RT-PCR of CXCL10 expression in Poly(I:C) with/or MS-275-treated MCs. The analysis was conducted as in **(A)**. Right: analysis of CXCL10 secretion from supernatants of MCs left untreated or pretreated with 0.25 μM of MS-275 for 48 (h) MCs were then treated with 2 ng/ml Poly(I:C) for 24 h alone or in the presence of MS-275. Cytokines were measured by an ELISA assay. **(D)** Cells were treated with 2 ng/ml Poly(I:C) for 3 h and/or 0.25 μM of MS-275 for 48 (h) Left: WB showing the phosphorylation status of P-STAT1 compared to STAT1 expression level in the total cellular extract of MCs. GAPDH was detected as a loading control. Right: WB quantification of phospo-STAT1 and total STAT1 protein levels in Poly(I:C) with/or MS-275-treated MCs compared to CTR. Bars represent the mean ± SEM of triplicate determinations in at least four independent experiments. *p*-value was calculated with respect to CTR or Poly(I:C) samples. Differences were considered significant: ^*^ *p* < 0.05; ^**^ *p* < 0.01; ns: not significant.

On the track of data obtained by proteomic analysis, the effect on type-I IFN response was further explored. The role of MS-275 in downregulating type I IFN response induced by Poly(I:C) was confirmed at both mRNA and protein expression levels. Antiviral target genes associated with IFNβ response, IFNβ, Mx1, IFIT1 and IFITM1, were downregulated by MS-275 treatment (**Figure 6A**). Western blot analysis confirmed the downregulation of Mx1, IFIT1, and IFITM1 (**Figure 6B**). Accordingly, secretion of CXCL10, an IFN-driven chemokine, was significantly decreased both as mRNA and as a protein (**Figure 6C**).

The observed impairment of the type I IFN response was functionally linked to reduced tyrosine phosphorylation of STAT1, a transcription factor with a central role in this pathway, upon MS-275-Poly(I:C) treatment (**Figure 6D**).

### Treatment with MS-275 differently modulates Poly(I:C)-induced proinflammatory cytokine/chemokine production

**Figure 7:**
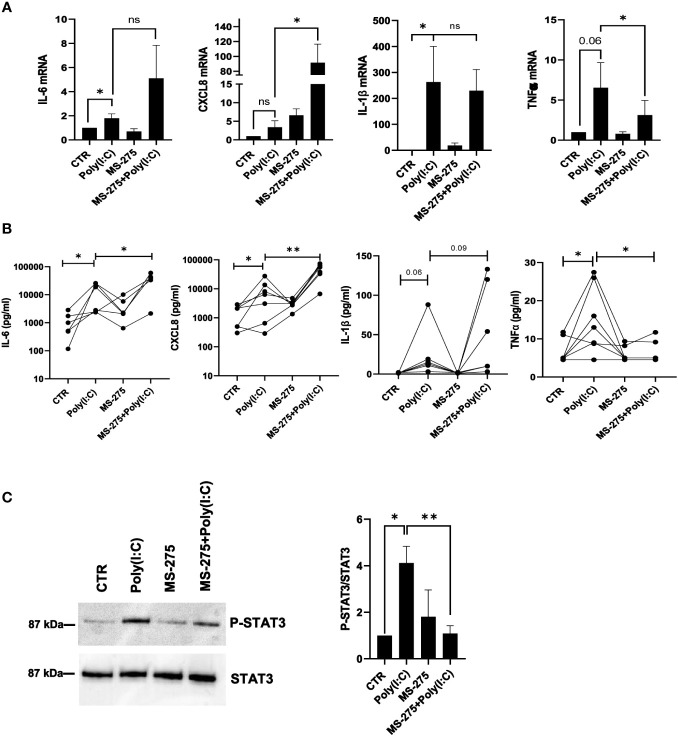
MS-275 differently modulates Poly(I:C)-induced proinflammatory cytokine/chemokine production. MCs were left untreated or pretreated with 0.25 μM of MS-275 for 48 (h) MCs were then treated with 2 ng/ml Poly(I:C) for 24 h alone or in the presence of MS-275. **(A)** Quantitative RT-PCR of *IL-6*, *CXCL8*, *IL-1β*, and *TNF-α* expression in Poly(I:C) with/or MS-275-treated MCs. *L34* mRNA levels were used for normalization. Bars represent the mean ± SEM of triplicate determinations in at least five independent experiments. **(B)** Analysis of TNF-α, IL-1β, IL-6, and CXCL8 secretion from supernatants of MCs left untreated or pretreated with 0.25 μM of MS-275 for 48 (h) MCs were then treated with 2 ng/ml Poly(I:C) for 24 h alone or in the presence of MS-275. Cytokine secretion was measured by an ELISA assay. **(C)** MCs were left untreated or pretreated with 0.25 μM MS-275 for 48 (h) MCs were then treated with 2 ng/ml Poly(I:C) for 3 h alone or in the presence of MS-275. Left: WB showing the STAT3 phosphorylation total cellular extract of MCs. STAT3 was detected as a loading control. Right: WB quantification of phospho-STAT3/STAT3 protein levels in Poly(I:C) with/or MS-275-treated MCs compared to CTR. Bars represent the mean ± SEM of triplicate determinations in at least five independent experiments. *p*-value was calculated with respect to CTR and Poly(I:C) samples. Differences were considered significant: ^*^ *p* < 0.05; ^**^ *p* < 0.01; ns: not significant.

The role of MS-275 in the production of inflammatory cytokines upon Poly(I:C) stimulation was then analyzed. MS-275 further increased Poly(I:C)-induced mRNA expression of IL-6 and CXCL8, but not of TNF-α and IL-1β. In particular, while TNF-α was significantly downregulated, the modulation of IL-1β showed great variability among samples (**Figure 7A**). MS-275 treatment significantly increased IL-6 and CXCL8 secretion, as shown by the ELISA assay. Instead, TNF-α secretion, but not IL-1β, was significantly decreased (**Figure 7B**). Interestingly, the reduced phosphorylation of STAT3, which is activated by many cytokines active in inflammation, including IL-6, IL-10, IL-11, and IL-21, suggests impaired cytokine signaling upon HDAC1-3 inhibition (Sulczewski et al., 2022) (**Figure 7C**).

Overall, these data demonstrate that HDAC1-3 inhibition by MS-275 is sufficient to modulate MMT induction upon Poly(I:C) stimulation, favoring the reacquisition of an epithelial-like phenotype. Moreover, MS-275 was sufficient to abolish the IFN response. Conversely, the effect on inflammatory cytokine production was not univocal and deserves further investigation.

## Discussion

Peritoneal fibrotic damage depends on complex interactions between external stimuli, intrinsic properties of the peritoneal membrane, and subsequent activation of the local innate-adaptive immune system (Terri et al., 2021). In this context, we studied the MC response to viral infection, focusing first on the role of TLR3, a sensor of virus-derived nucleic acids, in mediating changes in MC cell plasticity and the induction of an inflammatory response. The effects of TLR3 stimulation have been scarcely studied in MCs so far. TLR3 stimulation has been demonstrated to induce the expression of the matrix metalloproteinases/metalloproteinase inhibitors MMP9 and TIMP1, playing a role in ECM remodeling (Merkle et al., 2010). TLR3-mediated EMT and ECM remodeling in human small airway epithelial cells in a model of pulmonary chronic inflammation (Tian et al., 2017). TLR3 stimulation induced PAI-1 expression while downregulating t-PA in the mesothelium, controlling coagulation and fibrinolysis (Wornle et al., 2009a). Our data on TLR expression in human MCs from PD patients confirmed previous reports on MCs from human omentum, which were only partially reproduced in murine mesothelium (Hussain et al., 2008; Wornle et al., 2009b; Colmont et al., 2011). Interestingly, TLR3 stimulation induced the expression of other TLRs. In accordance with gene expression data, proteomic analysis showed an increased expression of TLR2 upon Poly(I:C) treatment, which was downregulated by MS-275. This observation was previously made in epithelial cells and suggests that viral sensing by TLR3 during influenza or SARS-CoV-2 infections may modulate the response toward other microorganisms, such as gram-negative and gram-positive bacteria during secondary infections, whose derivatives may activate TLR2 and TLR4 signaling (Takeuchi et al., 1999; Melkamu et al., 2013; Morris et al., 2017; Zhou et al., 2020). In MCs, treatment with Poly(I:C) promoted the induction of MMT-like features, such as the acquisition of a spindle-like shape, the downregulation of epithelial markers, and the upregulation of mesenchymal markers, including Snail. To our knowledge, no direct links have been established so far between TLR3 stimulation and MMT induction.

The cytokine/chemokine production analysis integrates previous reports obtained in murine and human MCs. The induction of CXCL8 expression by TLR3 activation was previously demonstrated in human MCs (Wornle et al., 2010). Moreover, TLR3 stimulation promoted the induction of a robust proinflammatory cytokine response in murine MCs (Park et al., 2007; Hwang et al., 2016).

When focusing on the molecular mechanisms underlying it, the effect of HDAC1-3 inhibition by MS-275 was analyzed. The effect of MS-275 has been studied especially in tumors, where it may provoke, depending on cellular systems, a variety of cellular effects, including a block of cell cycle progression, cell apoptosis, EMT reversal, block of tumor progression, angiogenesis, and metastasis (Naldi et al., 2009; Borbone et al., 2010).

Treatment of MCs with MS-275 was confirmed to revert the EMT/MMT state toward an epithelial-like phenotype, as previously demonstrated in biological contexts not related to viral infections (Shah et al., 2014; Rossi et al., 2018; Bontempi et al., 2022). The apparently paradoxical observation of induction of Snail in a frame of MMT reversal may be interpreted with the help of a previous study showing upregulated Snail expression coupled with functional inactivation upon MS-275 treatment in MCs (Rossi et al., 2018).

Previous proteomic analysis from malignant ascites of MS-275-treated mice injected with solid tumor cells revealed regulation of cell cycle, apoptosis, and senescence pathways (Du et al., 2022). Integration of single-cell sequencing and proteomic analysis revealed that MS-275, in combination with IL-2, favors the expression of inflammatory cytokines (Hicks et al., 2021).

On the track of proteomic analysis, we then focused on the type-I IFN response after Poly(I:C) treatment. HDAC1-3 inhibition downregulated type-I IFN response induced by treatment with Poly(I:C). In particular, Mx1, IFIT1, and IFITM1, targets of the IFN-β response, were strongly downregulated in MS-275-treated MCs. These observations confirm a previous study performed in HeLa cells, demonstrating that IFN-stimulated transcription requires HDAC activity (Chang et al., 2004). In detail, HDAC1 was demonstrated to associate with both STAT1 and STAT2 (Nusinzon and Horvath, 2003). We analyzed STAT1 activation status using a readout of STAT1 tyrosine phosphorylation, which was found to be reduced. An effect of MS-275 on STAT-1, but not STAT3 phosphorylation, was reported in T lymphocytes (Rosler et al., 2018). Indeed, MS-275 was demonstrated to shift STAT1 phosphorylation toward acetylation, blocking its activity (Xu et al., 2023). The reduced levels of CXCL10 were explained by the control exerted by both the type I IFN and IFN-γ pathways on the expression of this chemokine in fibroblasts (Ohta et al., 2014; Yu et al., 2020). Besides inhibiting type I response, MS-275 was recently demonstrated to favor SARS-CoV-2 infection in MCs by modulating ACE2 and TMPRSS2 expression (Trionfetti et al., 2023a). Overall, these results strongly suggest that treatment with HDAC1-3 inhibitor MS-275 may favor viral infection by multiple mechanisms.

We extended our analysis to inflammatory cytokines/chemokines. In this case, the results were nonunivocal; since IL-6 and CXCL8 were induced, IL-1β changes were not significant, whereas TNF-α secretion was inhibited upon Poly(I:C)/MS275 treatment.

HDAC1-3 inhibition by MS-275 was demonstrated to both induce and inhibit inflammatory cytokine production in different experimental systems. HDAC3 activity promoted TNF-α secretion in cardiomyocytes (Zhu et al., 2010). In accordance with this last study and our data, TNF-α production was reduced by MS-275 in response to *Candida albicans* and *Staphylococcus aureus* (Rosler et al., 2018). Interestingly, no data are available so far on the role of MS-275 in the production of inflammatory cytokines upon TLR3 stimulation.

MS-275 treatment also downregulated STAT3 tyrosine phosphorylation. This result apparently is not coherent with the increased levels of IL-6 (a STAT3 inducer) and of IL-6ST (an IL-6 receptor subunit) observed in the presence of MS-275. MS-275 was previously related to favoring STAT3 acetylation without affecting STAT3 phosphorylation (Shen et al., 2012; Rosler et al., 2018). This suggests that despite increased levels, IL-6 signaling is inhibited upon MS-275 treatment.

In conclusion, we provide evidence that MCs respond to Poly(I:C) undergoing MMT by inducing a type-I IFN response and secreting proinflammatory cytokines/chemokines. In this experimental setting, HDAC1-3 pharmacological inhibition by MS-275 was sufficient to promote a reversal of the MMT-like phenotype and to block the IFN response, which was linked to STAT1-reduced tyrosine phosphorylation. Conversely, treatment with MS-275 had no univocal effects on the expression of inflammatory cytokines/chemokines, since the secretion of IL-6 and CXCL8 was increased, whereas TNF-α and CXCL10 were decreased. Since HDAC-selective epidrugs, including MS-275, are currently being analyzed in clinical trials for the therapy of several tumors and have been proposed as tools to counteract nontumor fibrosis such as fibrotic disease in the pleura, kidney, and liver, the analysis of the complex molecular responses mediated by their action is relevant and deserves further study (Van Beneden et al., 2013; Korfei et al., 2022).

## Data availability statement

The original contributions presented in the study are included in the article/**Supplementary Material**, further inquiries can be directed to the corresponding author/s.

## Ethics statement

The studies involving humans were approved by Ethics Committee of Clinic Investigation of Sapienza University ref: 4697_2017 (Roma, Italy). The studies were conducted in accordance with the local legislation and institutional requirements. The participants provided their written informed consent to participate in this study.

## Author contributions

RS: Conceptualization, Funding acquisition, Supervision, Writing – original draft, Writing – review & editing. FT: Conceptualization, Data curation, Formal analysis, Investigation, Software, Validation, Writing – original draft. CM: Data curation, Formal analysis, Investigation, Methodology, Software, Writing – original draft. IC: Data curation, Formal analysis, Methodology, Writing – original draft. GB: Data curation, Investigation, Methodology, Software, Writing – original draft. MTe: Data curation, Investigation, Methodology, Writing – original draft. MTi: Data curation, Investigation, Methodology, Writing – review & editing. VM: Conceptualization, Investigation, Software, Writing – original draft. AD: Conceptualization, Data curation, Resources, Writing – original draft. PM: Resources, Supervision, Writing – original draft. MC: Funding acquisition, Supervision, Writing – original draft. CZ: Methodology, Resources, Writing – review & editing. GP: Data curation, Methodology, Supervision, Writing – review & editing. MR-O: Methodology, Supervision, Writing – review & editing. SV: Resources, Supervision, Writing – review & editing. AM: Methodology, Resources, Supervision, Writing – review & editing, Funding acquisition. MTr: Funding acquisition, Supervision, Writing – review & editing.
