# Supplementary figures and images for: Mechanisms of mesothelial cell response to viral infections: HDAC1-3 inhibition blocks poly(I:C)-induced type I interferon response and modulates the mesenchymal/inflammatory phenotype
Source: Front Cell Infect Microbiol. 2024 Feb 27;14:1308362. doi: 10.3389/fcimb.2024.1308362 (PMC10927979; doi:10.3389/fcimb.2024.1308362)

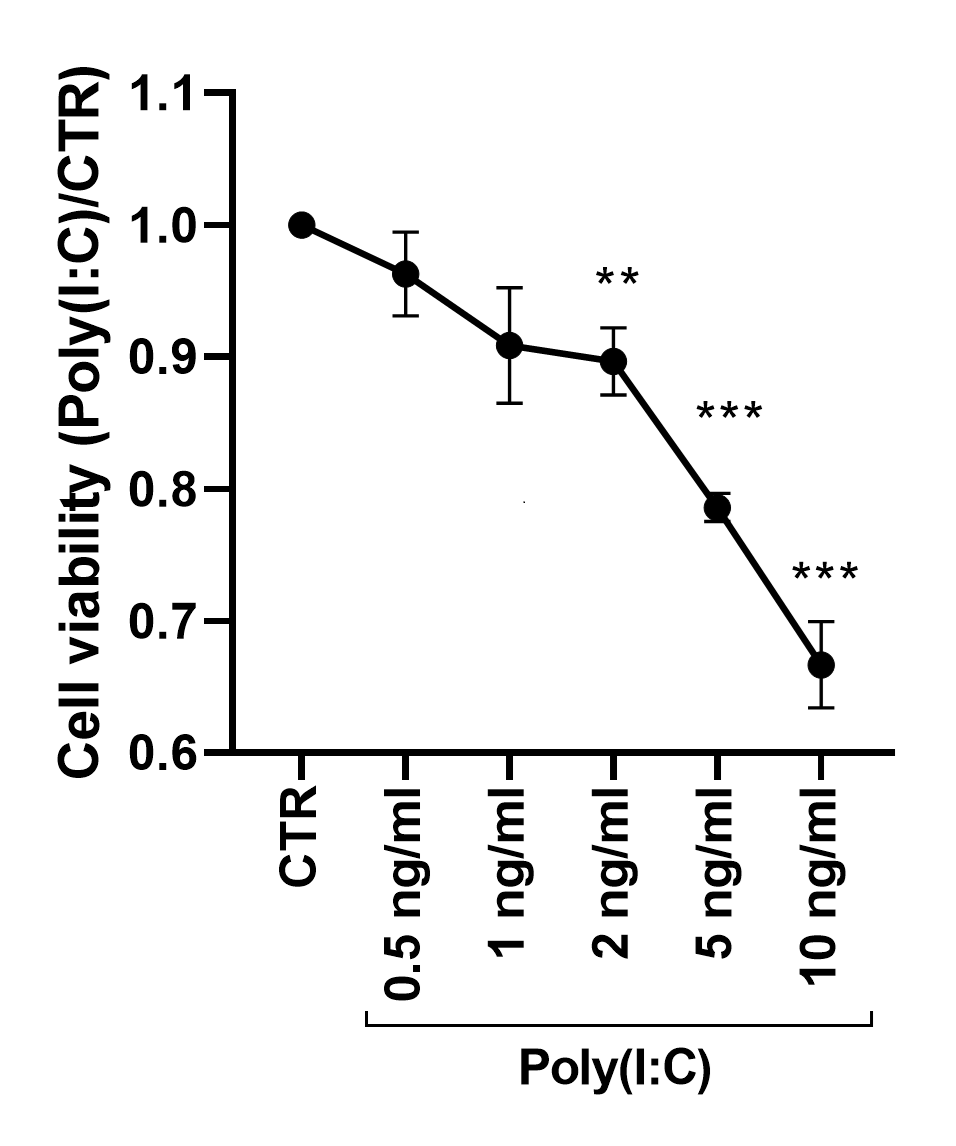

Supplement: Supplementary file 1 [file Image_1.png]
