# Supplementary material for: Mechanisms of mesothelial cell response to viral infections: HDAC1-3 inhibition blocks poly(I:C)-induced type I interferon response and modulates the mesenchymal/inflammatory phenotype
Source: Front Cell Infect Microbiol. 2024 Feb 27;14:1308362. doi: 10.3389/fcimb.2024.1308362 (PMC10927979; doi:10.3389/fcimb.2024.1308362)
Supplement: Supplementary file 5 [file DataSheet_4.docx]

**Supplementary Figure 1**: MTT viability assay on primary peritoneal MCs treated with Poly(I:C). MCs were treated for 24 hours with Poly(I:C) at different doses. Absorbance was measured at 480 nm and the results were folded on control (CTR) samples. Bars represent the mean ± SEM of triplicate determinations from nine independent experiments. P was calculated with respect to CTR. Differences were considered significant *: P < 0.05; ** P < 0.01; *** P < 0.001

**Supplementary Figure 2**: mass spectrometry data of modulated proteins in Ctr-Poly and Poly-Poly-275 comparisons resulted by volcano plot test (software Perseus). Identification and LFQ quantification features in addition to Gene Ontology terms and statistical significance of each identification were reported.

**Supplementary Figure 3**: mass spectrometry data of identified proteins belonging to Gene Ontology Biological Processes “inflammatory response” (GO: 0006954) (software Perseus). Identification and LFQ quantification features in addition to statistical significance of each identification were reported.

**Supplementary Figure 4**: GO biological processes statistically enriched in light blue cluster resulted by Hierarchical clustering performed using Euclidean distance and average linkage and highlighted in heat map visualization (software Perseus). Cluster size, number of proteins identified within the cluster and statistical significance related to each GO term were reported.
